# Tanshinone I Mitigates Steroid-Induced Osteonecrosis of the Femoral Head and Activates the Nrf2 Signaling Pathway in Rats

**DOI:** 10.1155/2021/8002161

**Published:** 2021-12-31

**Authors:** Xilin Xu, Yiwei Shen, Hang Lv, Jun Zhao, Xiaodong Li, Lu Gao, Shujun Ren, Xiaofeng Zhang

**Affiliations:** ^1^The Third Department of Orthopedics and Traumatology, The Second Affiliated Hospital of Heilongjiang University of Chinese Medicine, Harbin, Heilongjiang, China; ^2^Graduate School of Heilongjiang University of Chinese Medicine, Harbin, Heilongjiang, China; ^3^The First Department of Orthopedics and Traumatology, The Second Affiliated Hospital of Heilongjiang University of Chinese Medicine, Harbin, Heilongjiang, China; ^4^The Third Department of Orthopedics and Traumatology, First Affiliated Hospital, Heilongjiang University of Chinese Medicine, Harbin, Heilongjiang, China; ^5^Teaching and Research Section of Orthopedics and Traumatology, Heilongjiang University of Chinese Medicine, Harbin, Heilongjiang, China

## Abstract

Steroid-induced osteonecrosis of the femoral head (SIONFH) is a frequent orthopedic disease caused by long-term or high-dose administration of corticosteroids. Tanshinone I (TsI), a flavonoid compound isolated from *Salvia miltiorrhiza* Bunge, has been reported to inhibit osteoclastic differentiation *in vitro*. This study aimed to investigate whether TsI can ameliorate SIONFH. Herein, SIONFH was induced by intraperitoneal injection of 20 *μ*g/kg lipopolysaccharide every 24 h for 2 days, followed by an intramuscular injection of 40 mg/kg methylprednisolone every 24 h for 3 days. Four weeks after the final injection of methylprednisolone, the rats were intraperitoneally administrated with low-dose (5 mg/kg) and high-dose (10 mg/kg) TsI once daily for 4 weeks. Results showed that TsI significantly alleviated osteonecrotic lesions of the femoral heads as determined by micro-CT analysis. Furthermore, TsI increased alkaline phosphatase activity and expressions of osteoblastic markers including osteocalcin, type I collagen, osteopontin, and Runt-related transcription factor 2 and decreased tartrate-resistant acid phosphatase activity and expressions of osteoclastic markers including cathepsin K and acid phosphatase 5. TsI also reduced inflammatory response and oxidative stress and activated the nuclear factor erythroid 2-related factor 2 (Nrf2) signaling pathway in the femoral heads. Taken together, our findings show that TsI can relieve SIONFH, indicating that it may be a candidate for preventing SIONFH.

## 1. Introduction

Osteonecrosis of the femoral head (ONFH), a progressive bone disorder, causes subchondral bone microfractures and significant pain [1]. The age of patients ranged from 20 to 40 years [2]. Approximately 80% of patients receiving surgery during the early stages suffer from femoral heads collapse within 1–3 years [3]. The femoral heads collapse may rapidly progress to hip osteoarthritis that severely impacts hip joint function, ultimately undergoing artificial joint replacement [3]. At present, the long-term therapeutic effect of hip replacement is not usually ideal. Therefore, some patients may need to undergo 2-3 replacements, bringing an economic burden to patients and their families. If not treated promptly, patients with ONFH will suffer lifelong disability, which seriously affects the patients' quality of life. ONFH can be categorized as traumatic and nontraumatic. As nontraumatic ONFH, steroid-induced ONFH (SIONFH) was diagnosed in 26.84% of the ONFH patients in China, behind alcohol-induced ONFH [4]. Glucocorticoids are very effective at reducing inflammation, and they are commonly used for the treatment of systemic lupus erythematosus [5], rheumatoid arthritis [6], and multiple sclerosis [7]. Approximately 9%–40% of patients who receive long-term or high-dose (cumulative dose >3 g) glucocorticoid therapy develop ONFH [8, 9]. Therefore, the strategy to prevent ONFH during glucocorticoid therapy is urgently needed.

Dysregulation of bone homeostasis may be one of the leading causes of SIONFH. Following osteonecrosis, bone resorption mediated by osteoclasts surpasses bone formation by osteoblasts, leading to loss of subchondral trabecular bone. Elevated levels of proinflammatory cytokines have been found in SIONFH patients [10]. It is widely accepted that proinflammatory cytokines interfere with bone homeostasis [11]. In addition, glucocorticoids have a significant influence on oxidative stress [12]. Excessive oxidative stress leads to an imbalance between bone resorption and bone formation, thereby leading to loss of bone mineral density [13]. Multiple drugs exerted therapeutic effects on ONFH through their antioxidative properties [14–16], highlighting the significance of oxidative stress in the pathogenesis of ONFH.

Tanshinone I (TsI) is one of the major bioactive flavonoids isolated from *Salvia miltiorrhiza* Bunge (also known as Danshen in Chinese), which has a tremendous potential for treating cardiovascular diseases [17]. TsI exerts antitumor [18], anti-inflammatory [19], and antioxidative [20] effects on different cell types. Emerging studies have shown that Danshen is a component frequently used in Chinese herbal formulas, including Huo-gu and Fufang Xian Ling Gu Bao, which are effective in preventing SIONFH [21, 22]. As one of the most abundant lipophilic compounds in Danshen, TsI has a significant effect on inhibiting osteoclast differentiation [23, 24]. As abovementioned, osteoclasts induce bone resorption that plays a pivotal role in the progression of ONFH. Therefore, it is worthy of discussion whether TsI has a protective effect against ONFH.

Here, SIONFH was induced in rats by injection of lipopolysaccharide (LPS) and methylprednisolone (MPS), and the rats received TsI treatment for 4 weeks. Subsequently, the effect of TsI on the osteogenesis, osteoclastogenesis, inflammatory response, oxidative stress, and the nuclear factor erythroid 2-related factor 2 (Nrf2) signaling pathway in the SIONFH rats was analyzed. This study provides a potential candidate for SIONFH treatment.

## 2. Materials and Methods

### 2.1. Ethics Statement

Animal experiments were strictly in accordance with the Guide for the Care and Use of Laboratory Animals. The protocol of animal experiments in this study was approved by the Institutional Animal Care and Use Committee of the Heilongjiang University of Chinese Medicine.

### 2.2. Experimental Animals and Grouping

Male 12-week-old Sprague Dawley (SD) rats were obtained from Beijing HFK Bioscience (China). Rats were maintained at 22 ± 1°C with relative humidity at 45%–55% and a 12/12 h light/dark cycle and allowed free access to food and water. After 7-day feeding adaptation, all the rats were randomized into 4 groups: the control group, the SIONFH group, the SIONFH + low-dose TsI (TsI-L) group, and the SIONFH + high-dose TsI (TsI-H) group.

### 2.3. Induction of SIONFH and Administration of TsI

Rats in the SIONFH group, the SIONFH + TsI-L group, and the SIONFH + TsI-H group were intraperitoneally injected with lipopolysaccharide (LPS, Sigma-Aldrich, USA) at a concentration of 20 *μ*g/kg body weight on day 0 and day 1 at a time interval of 24 h, followed by intramuscular injection with methylprednisolone (MPS, Pfizer bio, China) at a concentration of 40 mg/kg body weight on day 3, day 4, and day 5 at a time interval of 24 h as previously described [25]. Rats in the control group only received the same volume of normal saline on day 0, day 1, day 3, day 4, and day 5.

TsI (Aladdin, China) was dissolved in DMSO, followed by dilution with normal saline. Rats in the SIONFH + TsI-L group and the SIONFH + TsI-H group were intraperitoneally injected with TsI at a concentration of 5 mg/kg and 10 mg/kg body weight once daily for 4 weeks after the final injection of MPS, respectively. Rats in the control group and the SIONFH group received the same volume of vehicle. After 4 weeks, all the rats were euthanized, and the bilateral femora and serum were harvested for the following experiments.

### 2.4. Micro-CT Scan and Quantitative Analysis

The femoral heads were subjected to micro-CT scanning using the Quantum GX2 micro-CT (PerkinElmer, USA). Femoral heads on the right side of each group were scanned at 90 kV/88 *μ*A of energy with an entire scan length of 5 mm at a 10 *μ*m pixel size. Bone mineral density (BMD), the ratio of bone volume/total volume (BV/TV), trabecular number (Tb.N), trabecular separation (Tb.Sp), and trabecular thickness (Tb.Th) were quantified.

### 2.5. Alkaline Phosphatase (ALP) and Tartrate-Resistant Acid Phosphatase (TRAP) Activity Assay

The activity of ALP in the femoral head and that of TRAP in the serum were measured using the ALP assay kit (Nanjing Jiancheng, China) and the TRAP assay kit (Nanjing Jiancheng, China), respectively, in accordance with their manufacturer's instructions.

### 2.6. Real-Time Quantitative PCR (RT-qPCR)

TRIpure (BioTeke, China) was used to isolate total RNA from the femoral heads. Complementary DNA was then synthesized from the total RNA by using BeyoRT II M-MLV reverse transcriptase (D7160L, Beyotime, China). RT-qPCR was performed with SYBR Green (Solarbio, China). Primer sequences were listed as follows: osteocalcin (OCN) forward primer 5′-GGCAGTAAGGTGGTGAATAG-3′, OCN reverse primer 5′-GTCCTGGAAGCCAATGTG-3′; type I collagen (COL I) forward primer 5′-CGAGTATGGAAGCGAAGGT-3′, COL I reverse primer 5′-CCACAAGCGTGCTGTAGGT-3′; osteopontin (OPN) forward primer 5′-CTTGGCTTACGGACTGA-3′, OPN reverse primer 5′-AACTGGGATGACCTTGAT-3′; runt-related transcription factor 2 (Runx2) forward primer 5′-CCCAACTTCCTGTGCTCC-3′, Runx2 reverse primer 5′-AGTGAAACTCTTGCCTCGTC-3′; tumor necrosis factor-*α* (TNF-*α*) forward primer 5′-CGGAAAGCATGATCCGAGAT-3′, TNF-*α* reverse primer 5′-AGACAGAAGAGCGTGGTGGC-3′; interleukin-6 (IL-6) forward primer 5′-CAGCCACTGCCTTCCCTA-3′, IL-6 reverse primer 5′-TTGCCATTGCACAACTCTTT-3′; and interleukin-1*β* (IL-1*β*) reverse primer 5′-TTCAAATCTCACAGCAGCAT-3′, IL-1*β* forward primer 5′-CACGGGCAAGACATAGGTAG-3′.

### 2.7. Western Blot Analysis

Total protein was isolated from the femoral head with RIPA lysis buffer (Beyotime Biotech, China) containing 1% PMSF (Beyotime Biotech). Nuclear protein was isolated using the nuclear and cytoplasmic protein extraction kit (Beyotime Biotech) according to the manufacturer's protocols. The protein samples (15–30 *μ*g per lane) were separated by sodium dodecyl sulfate-polyacrylamide gel electrophoresis, transferred onto a PVDF membrane (ThermoFisher Scientific, USA), and blocked with 5% BSA (Biosharp Life Sciences, China) in Tris-buffer saline containing 0.15% Tween-20. The membranes were probed overnight at 4°C with primary antibodies including mouse monoclonal anti-*β*-actin (60008-1-Ig, 1 : 2000) antibody and rabbit polyclonal anti-histone H3 (17168-1-AP, 1 : 500), anti-OCN (DF12303, 1 : 1000), anti-COL I (AF0134, 1 : 1000), anti-OPN (AF0227, 1 : 1000), anti-Runx2 (AF5186, 1 : 1000), anti-cathepsin K (CTSK, DF6614, 1 : 1000), anti-acid phosphatase 5 (ACP5, DF6989, 1 : 1000), anti-heme oxygenase-1 (HO-1, AF5393, 1 : 1000), anti-NADPH quinone dehydrogenase 1 (NQO1, DF6437, 1 : 1000), and anti-Nrf2 (AF0639, 1 : 1000) antibodies. The anti-*β*-actin and anti-histone H3 antibodies were purchased from Proteintech Group, Inc. (USA), and the other primary antibodies were purchased from Affinity Biosciences (China). After being washed, the membranes were incubated with horseradish peroxide-conjugated goat anti-mouse (SA00001-1, 1 : 10000) or goat anti-rabbit (SA00001-2, 1 : 10000) secondary antibodies (Proteintech) for 40 min at 37°C. *β*-Actin and histone H3 were used as a loading control. The protein bands were visualized with the ECL system (7 Sea Biotech, China).

### 2.8. Immunohistochemistry (IHC) Staining

IHC staining was performed using rabbit polyclonal anti-OCN (DF12303) and anti-COL I (AF0134) antibodies (Affinity Biosciences). In brief, the femoral heads were decalcified, embedded with paraffin, and sectioned at a thickness of 5 *μ*m in the coronal plane. The sections were immersed in antigen retrieval solution (1.8% citric acid and 8.2% sodium citrate) for 10 min and then in 3% hydrogen peroxide for 15 min to block endogenous peroxidase activity. After blocking in goat serum (Solarbio) for 15 min, the sections were incubated overnight at 4°C with anti-OCN or anti-COL I antibody (1 : 50), followed by incubation with horseradish peroxide-conjugated goat anti-rabbit antibody (#31460, Thermo Fisher Scientific, 1 : 500) for 60 min at 37°C. The sections were stained with DAB (Solarbio) and counterstained with hematoxylin (Solarbio). The pictures were captured using a microscope (×100) (OLUMPUS, Japan).

### 2.9. Enzyme-Linked Immunosorbent Assay (ELISA)

The concentration of proinflammatory factors including TNF-*α*, IL-6, and IL-1*β* in the serum was determined by commercially available ELISA kits (MultiSciences Biotech, China) in accordance with their manufacturer's protocols.

### 2.10. Superoxide Dismutase (SOD), Glutathione Peroxidase (GSH-Px), and Chloramphenicol Acetyltransferase (CAT) Activity Assay

The activity of antioxidant enzymes including GSH-Px, SOD, and CAT in the femoral head was detected using commercial assay kits (Nanjing Jiancheng) following their manufacturer's instructions.

### 2.11. Statistical Analysis

Data are presented as the mean ± standard deviation (SD). Statistical analyses were performed using GraphPad Prism (version 8.0.1, GraphPad Software, USA) by the Kruskal–Wallis test (Tb.N) with Dunn's test or one way ANOVA (other data) with Tukey's test. The *p* values less than 0.05 were taken to be statistically significant.

## 3. Results

### 3.1. SIONFH Is Attenuated by TsI in the MPS-Induced Rat Model

**Figure 1:**
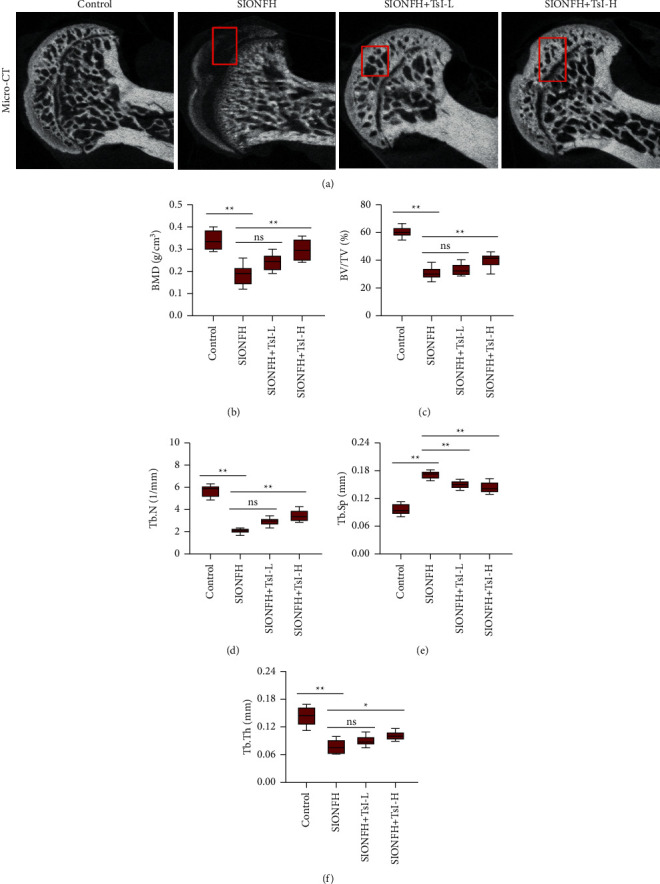
Micro-CT scans and analyses of the femoral heads of the MPS-induced SIONFH rat model. (a) Representative micro-CT images of the femoral heads (coronal images). Micro-CT images showed significantly less subchondral trabeculae in the SIONFH group compared with that in the control group, while more trabeculae were found in the SIONFH + TsI-L group and the SIONFH + TsI-H group. (b–f) Quantitative analyses for microstructural parameters of trabecular bone including BMD, BV/TV, Tb.N, Tb.Sp, and Tb.Th. ^*∗*^*p* < 0.05; ^*∗∗*^*p* < 0.01; ns, no significant. BMD, bone mineral density; BV/TV, bone volume per tissue volume; Tb.N, trabecular number; Tb.Sp, trabecular separation; Tb.Th, trabecular thickness.

To evaluate the influence of TsI on the rat model of SIONFH, SIONFH was induced by intraperitoneal injection of LPS and intramuscular injection of MPS according to a previous study [25], followed by intraperitoneal injection of low-dose or high-dose TsI. After 4 weeks of administration of TsI, micro-CT scanning was employed to analyze the bone structure of the femoral heads. As shown in Figure 1(a), the femoral heads in the SIONFH group appeared to have obvious collapse of the subchondral area, indicating osteonecrosis developed in the rats of the SIONFH group. However, TsI treatment attenuated the MPS-induced osteonecrosis in the femoral heads (Figure 1(a)). The microarchitectural parameters including BMD, BV/TV ratio, Tb.N, Tb.Sp, and Tb.Th were quantified (Figures 1(b)–1(f)). The values of these parameters in the SIONFH group (BMD, 0.19 ± 0.04 g/cm^3^; BV/TV ratio, 30.66% ± 4.17%; Tb.N, 2.07 ± 0.21/mm; Tb.Sp, 0.17 ± 0.01 mm; and Tb.Th, 0.08 ± 0.01 mm) were significantly lower than those in the control group (BMD, 0.34 ± 0.04 g/cm^3^; BV/TV ratio, 60.45% ± 3.57%; Tb.N, 5.71 ± 0.50/mm; Tb.Sp, 0.10 ± 0.01 mm; and Tb.Th, 0.14 ± 0.02 mm). Both low-dose and high-dose TsI treatments significantly prevented MPS-induced increases in the value of Tb.Sp (SIONFH + TsI-L group, 0.15 ± 0.01 mm; SIONFH + TsI-H group, 0.14 ± 0.01 mm). Other parameters were only improved by high-dose TsI treatment (BMD, 0.30 ± 0.04 g/cm^3^; BV/TV ratio, 40.11% ± 4.98%; Tb.N, 3.43 ± 0.49/mm; and Tb.Th, 0.10 ± 0.01 mm).

### 3.2. TsI Facilitates Osteogenesis in the SIONFH Rat Model

**Figure 2:**
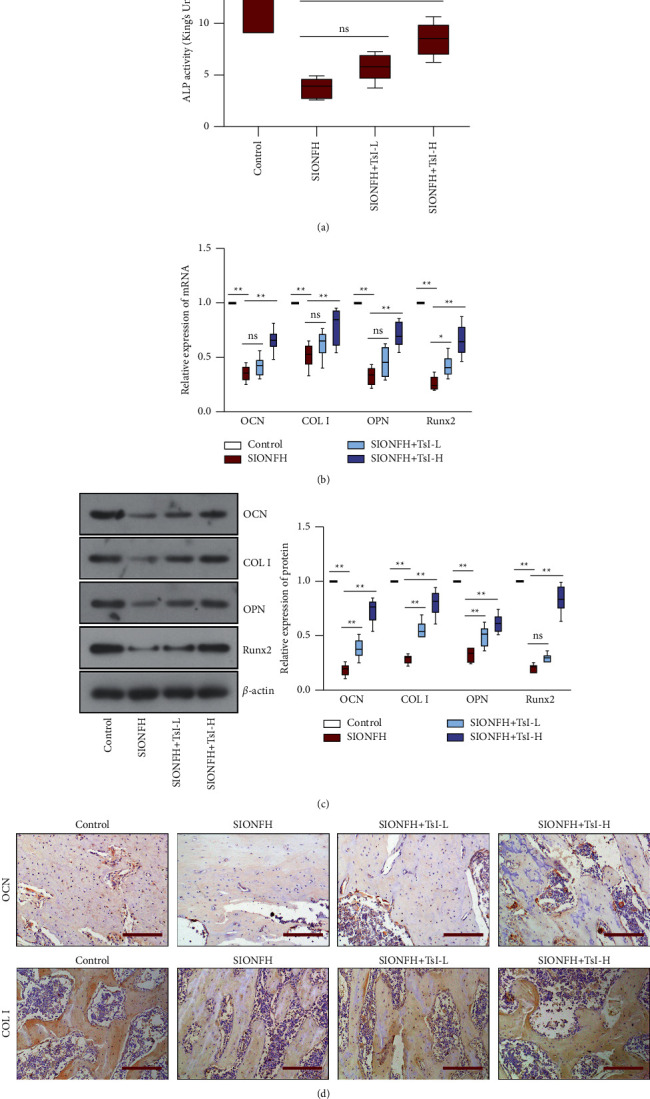
TsI promotes osteogenesis in the femoral heads of the MPS-induced SIONFH rat model. (a) ALP activity in the femoral heads. (b) mRNA levels of OCN, COL I, OPN, and Runx2 in the femoral heads. (c) Expression levels of OCN, COL I, OPN, and Runx2 in the femoral heads. (d) Representative images of OCN staining and COL I staining (scale bar, 200 *μ*m) in the femoral heads. ^*∗*^, *p* < 0.05; ^*∗∗*^, *p* < 0.01; ns, no significant. ALP, alkaline phosphatase; OCN, osteocalcin; COL I, type I collagen; OPN, osteopontin; Runx2, Runt-related transcription factor 2.

Next, the effect of TsI on osteogenesis in the rat model of SIONFH was explored. The SIONFH group (3.86 ± 1.05 King's unit/g prot) had a low level of ALP activity compared with the control group (11.95 ± 2.53 King's unit/g prot); however, high-dose TsI partly diminished the SIONFH-induced decreases in ALP activity (8.53 ± 1.75 King's unit/g prot) (Figure 2(a)). The expression of osteogenesis makers including OCN, COL I, OPN, and Runx2 was detected by RT-qPCR and western blot analysis. Results exhibited that the mRNA and protein expression levels of OCN, COL I, OPN, and Runx2 were significantly decreased in the SIONFH group (Figures 2(b), 2(c)). High-dose TsI treatments upregulated the mRNA and protein expression of all these osteogenesis makers in the femoral heads of SIONFH rats, while low-dose TsI treatments only upregulated the mRNA expression of Runx2 and the protein expression of OCN, COL I, and OPN (Figures 2(b), 2(c)). Consistently, the increases of OCN and COL I expression by low-dose and high-dose TsI treatments in the femoral heads were also confirmed by IHC staining (Figure 2(d)). These results indicated that TsI could promote bone formation in the rat model of SIONFH.

### 3.3. TsI Reduces Osteoclastogenesis in the SIONFH Rat Model

**Figure 3:**
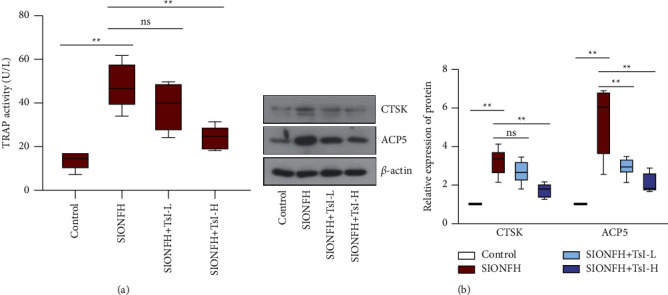
TsI restrains osteoclastogenesis in the femoral heads of the MPS-induced SIONFH rat model. (a) TRAP activity in the serum. (b) Expression levels of CTSK and ACP5 in the femoral heads. ^*∗∗*^*p* < 0.01; ns, no significant. TRAP, tartrate-resistant acid phosphatase; CTSK, cathepsin K; ACP5, acid phosphatase 5.

To analyze whether TsI affected osteoclastogenesis in the rat model of SIONFH, the activity of serum TRAP and the expression of CTSK and ACP5 were measured. Elevated activities of serum TRAP were observed in the SIONFH group (the SIONFH group, 47.62 ± 10.30 U/L; the control group, 13.66 ± 3.84 U/L), which could be inhibited by high-dose TsI treatment (24.20 ± 5.42 U/L) (Figure 3(a)). Western blot analysis showed that the expression levels of CTSK and ACP5 in the femoral heads were upregulated after induction of SIONFH, and this action was reversed by high-dose TsI, while low-dose TsI only suppressed the upregulation in ACP5 expressions (Figure 3(b)). These results indicated that TsI could inhibit bone resorption in the rat model of SIONFH.

### 3.4. TsI Suppresses Inflammatory Response in the SIONFH Rat Model

**Figure 4:**
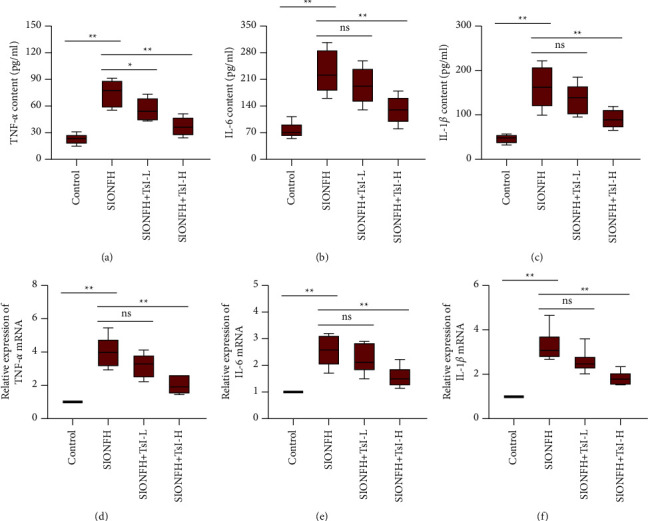
TsI suppresses the inflammatory response in MPS-induced SIONFH rats. (a–c) Contents of TNF-*α*, IL-6, and IL-1*β* in the serum. (d–f) mRNA levels of TNF-*α*, IL-6, and IL-1*β* in the femoral heads. ^*∗*^*p* < 0.05; ^*∗∗*^*p* < 0.01; ns, no significant. TNF-*α*, tumor necrosis factor alpha; IL-6, interleukin-6; IL-1*β*, interleukin-1*β*.

To determine whether TsI influenced the inflammatory response in the SIONFH rat model, the level of proinflammatory cytokines including TNF-*α*, IL-6, and IL-1*β* in the serum was analyzed using ELISA (Figures 4(a)–4(c)). Compared with the control group (TNF-*α*, 23.34 ± 5.61 pg/ml; IL-6, 76.61 ± 20.17 pg/ml; IL-1*β*, 46.41 ± 9.94 pg/ml), the SIONFH group has a higher level of proinflammatory cytokines (TNF-*α*, 74.99 ± 15.00 pg/ml; IL-6, 229.72 ± 55.35 pg/ml; IL-1*β*, 162.37 ± 47.10 pg/ml). High-dose TsI significantly inhibited the increase of those cytokines (TNF-*α*, 37.36 ± 10.26 pg/ml; IL-6, 130.23 ± 35.54 pg/ml; IL-1*β*, 91.35 ± 20.85 pg/ml), while low-dose TsI only significantly inhibited the increases in the level of TNF-*α* (56.10 ± 12.48 pg/ml) (Figure 4(a)). The mRNA expression of these proinflammatory cytokines was detected by RT-qPCR. Increased expression of TNF-*α*, IL-6, and IL-1*β* was observed in the SIONFH group, which was inhibited by high-dose TsI (Figures 4(d)–4(f)). These results indicated that TsI could suppress inflammatory responses in the rat model of SIONFH.

### 3.5. TsI Neutralizes Oxidative Stress in the SIONFH Rat Model

**Figure 5:**
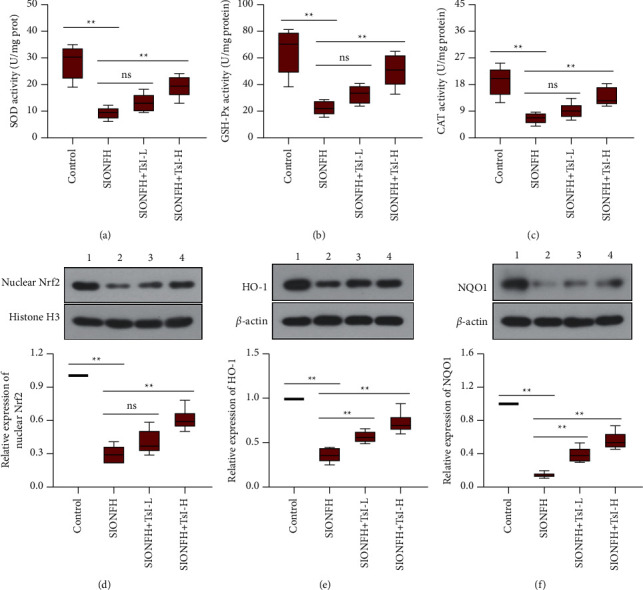
TsI inhibits oxidative stress and activates the Nrf2 signaling pathway in the MPS-induced SIONFH rat model. (a–c) SOD, GSH-Px, and CAT activities in the femoral heads. (d–f) Expression levels of nuclear Nrf2, HO-1, and NQO1 in the femoral heads. 1, the control group; 2, the SIONFH group; 3, the SIONFH + TsI-L group; 4, the SIONFH + TsI-H group. ^*∗∗*^*p* < 0.01; ns, no significant. SOD, superoxide dismutase; GSH-Px, glutathione peroxidase; CAT, chloramphenicol acetyltransferase; Nrf2, nuclear factor erythroid 2-related factor 2; HO-1, heme oxygenase; NQO1, NADPH quinone dehydrogenase 1.

The protective effect of TsI against oxidative stress in the femoral heads was also assessed, and the results showed the decreased activity of GSH-Px (22.16 ± 5.32 U/mg prot), SOD (9.33 ± 2.18 U/mg prot), and CAT (6.70 ± 1.95 U/mg prot) in the SIONFH group compared with those of the control group (GSH-Px, 64.82 ± 16.67 U/mg prot; SOD, 28.51 ± 6.24 U/mg prot; CAT, 19.22 ± 4.96 U/mg prot). The decreased activity of those enzymes was partly restored by high-dose TsI treatment (Figures 5(a)–5(c); GSH-Px, 50.24 ± 11.87 U/mg prot; SOD, 19.20 ± 3.99 U/mg prot; CAT, 13.73 ± 3.05 U/mg prot). The proteins involved in the Nrf2 pathway including Nrf2, HO-1, and NQO1 were detected using western blot analysis. The expression of Nrf2, HO-1, and NQO1 was significantly downregulated in the femoral heads after induction of SIONFH, but high-dose TsI treatment reversed the downregulation of expression of these proteins (Figures 5(d)–5(f)). These results indicated that TsI could reduce oxidative stress and activate the Nrf2 signaling pathway in the rat model of SIONFH.

## 4. Discussion

As early as 1932, the detrimental effect of hypercortisolism on bone was identified [26]. With the extensive application of glucocorticoids in clinics, over 50% of patients who receive glucocorticoid therapy develop significant bone loss that results from dysregulation of bone metabolism [27]. Although the detailed mechanisms implicated in the pathogenesis of SIONFH are not fully understood, it is generally indicated that the impairment of the imbalance between bone formation and bone resorption may be one of the most common biological processes in SIONFH.

As a traditional herbal medicine in China, Danshen is frequently used to treat some different types of diseases including ONFH. Flavonoids are the major chemical constituents of many traditional herbal medicines, including *Scutellaria baicalensis* (Huangqin), *Sophora flavescens* (Kushen), *Puerariae Lobatae* Radix (Gegen), and *Carthamus tinctorius* (Hong Hua). Accumulating evidence has demonstrated that lots of flavonoids are effective to treat ONFH, including naringin [28], icariin [29], and luteolin [30]. TsI is one of the main active components in Danshen and its antiosteoclastic effect has been reported in *in vitro* experiments in earlier studies [23, 24]. In the present study, we found that TsI could attenuate SIONFH in a rat model. Moreover, TsI was proved to stimulate osteogenesis and inhibit osteoclastogenesis, indicating its potentials in restoring the balance between bone formation and bone resorption.

Emerging studies have shown that the level of proinflammatory cytokines is elevated in the serum of SIONFH patients [10]. Generally, glucocorticoid treatment is given to suppress inflammation. However, long-term or excess application of glucocorticoids functions as an activator for inflammation. The elevation of proinflammatory cytokines has been found in a mouse model of SIONFH [10, 31]. Similar results were found in the rat model of SIONFH in the current study. We also found that TsI exerted an anti-inflammatory effect in the rat model of SIONFH. Bone homeostasis can be critically affected by inflammation. Several earlier studies confirmed that TNF-*α*, IL-1*β*, and IL-6 had negative effects on osteoblast differentiation [32–34]. TNF-*α* and IL-1*β* have also been reported to stimulate osteoclast differentiation [35, 36]. Therefore, the regulatory role of TsI in bone formation and bone resorption may be ascribed to its anti-inflammatory potential.

Oxidative stress develops when the balance between reactive oxygen species (ROS) generation and ROS scavenging is broken. Antioxidant enzymes are involved in ROS scavenging. It has been reported that oxidative stress is involved in the pathogenesis of multiple bone diseases including ONFH [37–39]. ROS has a negative effect on bone homeostasis due to its pro-osteoclastic effects [40]. Oxidative stress causes dysfunction of bone metabolism-related cells including bone mesenchymal stem cells [41], osteoblasts [42], osteoclasts [43], and osteocytes [44]. Glucocorticoids stimulate overproduction of ROS by depletion of antioxidant molecules or inhibition of antioxidant enzymes. Excessive usage of glucocorticoids has been indicated to enhance oxidative stress in an animal model of SIONFH [45]. The function of TsI in inhibiting oxidative stress has been reported in several studies [20]. Consistently, this study observed that TsI exerted its antioxidant potential in SIONFH by increasing the activities of antioxidant enzymes. Considering the important role of oxidative stress in bone homeostasis, the protective effect of TsI against SIONFH may be attributable to its antioxidant potential. In addition, oxidative stress is a pivotal factor in the inflammatory response. Excessive accumulation of ROS aggravates inflammatory response via activation of the nuclear factor-*κ*B (NF-*κ*B) signaling pathway in the development of many diseases [46, 47]. Therefore, the anti-inflammatory potential of TsI may be associated with its antioxidant potential, which needs to be confirmed in the subsequent works.

The transcription factor Nrf2 plays a central role in the maintenance of intracellular redox homeostasis. Stabilized Nrf2 translocates into the nucleus, where it binds to antioxidant-response elements (ARE) in the promoter region of Nrf2 target genes to activate downstream antioxidant enzymes [48]. Accumulating investigations have indicated that the Nrf2 signaling pathway is inhibited in necrotic femoral head tissues and the ONFH model [49]. The present study also confirmed the findings. Earlier findings demonstrated that TsI could activate the Nrf2 signaling [50]. Similarly, we found that TsI activated the Nrf2 signaling in the femoral head of SIONFH models. The increased activities of antioxidant enzymes by TsI may be associated with activation of the Nrf2 signaling. In addition, Nrf2 serves as an enhancer of osteogenesis [51] and a suppressor of osteoclastogenesis [52]. Therefore, TsI might promote osteogenesis and restrain osteoclastogenesis through the activation of the Nrf2 signaling pathway in the femoral heads.

It is a limitation that there is no positive control drug in the present study. Although this study has shown the effect of TsI against SIONFH, it is hard to declare to what extent TsI has protective effects. Therefore, a comparison of the therapeutic efficacy of TsI with a positive control drug will be needed in our future study.

## 5. Conclusion

In summary, our results revealed that TsI could promote osteogenesis and inhibit osteoclastogenesis in the femoral heads of SIONFH rats. Moreover, TsI exerted both anti-inflammatory and antioxidant potential in the SIONFH rats. In addition, TsI activated the Nrf2 signaling pathway. These findings suggest that TsI may be a good candidate for treating SIONFH.

## Data Availability

The datasets generated during and/or analyzed during the current study are available from the corresponding author on reasonable request.
